# Ymr210wp leads to the accumulation of phospholipids and steryl esters in yeast

**DOI:** 10.6026/97320630013360

**Published:** 2017-11-30

**Authors:** Naresh Kumar Manda, Venkata Bala Sai Chaitanya Thunuguntla, Chandrasekhar Bokka, Bondili Jayakumar Singh

**Affiliations:** 1 Department of Biotechnology, K L University, Green fields, Vaddeswaram, Guntur Dist, Andhra Pradesh, India - 522502

**Keywords:** TAG Lipase, Ester Hydrolase, Lipid droplets, Saccharomyces cerevisiae, Triacylglycerols, Steryl esters

## Abstract

Ymr210w was identified as a MAG (Monoacylglycerol) lipase. The accumulation of the phospholipids in the ymr210wΔ was not clearly understood. It was expressed in S. cerevisiae using pYES2/CT vector and His-tag purified recombinant protein confirmed TAG lipase activity. To further evaluate the role of YMR210w, ester hydrolase activity was also confirmed with pNP-acetate, pNP-butyrate and pNP - palmitate. GC-MS lipid profiling of ymr210wΔ showed an increase in the 15:0 Pentadecanoic acid by 76% among the total lipids. Phospholipid, Erucic acid 22:1 (Δ13) showed 43% increase while steryl esters showed significant changes with 16:0 hexadecanoic acid augmentations by 80% and 18:0 Octadecanoic acid by 165% when compared to wild type (WT). Increase in the steryl ester and TAG content supports the accumulation of lipid bodies in ymr210wΔ strain when compared with WT cells.

## Background

Yeast lipases and ester hydrolases involved in lipid metabolism play a major role in signaling, cell cycle and survival. Most of the lipase enzymes were found to be multifunctional in Saccharomyces cerevisiae. Tgl3p, the lipid droplets localized TAG lipase exhibited DAG hydrolysis activity along with definite lyso phosphotidyl ethanolamine (LPE) Acyl transferase activity in vitro [1, 2, 3]. Following this, Tgl4p and Tgl5p were reported with the conserved lipase motif [4]. Tgl4p is the major multifunctional enzyme involved in lipid metabolism. Besides TAG lipase activity Tgl4p also showed calcium independent phospholipase PLA2 activity and LPA acyl transferase activity [5]. In addition to Tgl3p, Tgl5p also displayed HXXXXD motif but preferentially acted as lysophosphatidicacid (LPA) Acyl tranferase [2, 3, 4].

Earlier, YMR210w was mentioned as a member of EHT1 and EEB1 gene clad. Even though, it was found to be redundant and involved in medium chain fatty acid ethyl ester synthesis, production of ethyl Octanoate and ethyl deconoate only in the absence of EHT1 and EEB1 genes [6]. Ymr210wp known as a MAG lipase [7]. It was shown that over expression of Ymr210w decreases TAG levels. Under homeostatic conditions, it was attributed that the reduction in TAG levels is due to lack of required quantity of MAG as precursor for TAG synthesis. But, the FFAs (Free Fatty Acids) released by the MAG lipase activity of Ymr210wp were not clearly shown. Further, there was no phosholipase or lysophospholipase activity found but the concentration of PC and PE were also found to be increased in YMR210w Δ [7]. Further to understand the role of YMR210w in lipid metabolism, enzymatic assays and GC MS based lipid profile characterization of WT and YMR210w Δ were performed. This study highlights the dual functionality of Ymr210wp as ester hydrolase along with TAG lipase activity and showed accumulation of steryl esters and phospholipids.

## Methodology

### Yeast strains and growth conditions

Strains used in this study are Saccharomyces cerevisiae BY4741 (WT), YMR210w over expressed in WT (OE), ymr210wΔ and only Vector pYES2/CT cloned in WT (V). WT and ymr210wΔ cells were grown either in YPD medium containing 1% yeast extract, 2% peptone and 2% dextrose weight/volume (w/v) or synthetic minimal medium (SC+Ura) containing 0.67% yeast nitrogen base (YNB), supplemented with the complete supplement mixture 0.192% appropriate amino acids without uracil, 2% dextrose and 0.015% uracil (w/v). Recombinant yeast strains i.e., OE and V were cultured in synthetic minimal medium (SC-Ura) containing 0.67% YNB, supplemented with the complete supplement mixture 0.192% appropriate amino acids without uracil and 2% dextrose. Induction was done in SC-Ura media with 2% raffinose and 3xYP medium with 6% galactose. All cells were cultured in liquid media at 30°C and 180 rpm.

### Cloning and expression of the recombinant YMR210w

YMR210w was cloned into pYES2/CT vector and transformed into DH5α cells. Only vector and vector plus construct were transformed into WT individually by using the Frozen-EZ Yeast Transformation kit (Zymo Research, USA) following the manufacturer's protocol. Expression of the Recombinant YMR210w in WT and V was performed as per Gelperin DM et al. (2005) [8].

### Esterase assay using p-Nitrophenyl ester substrates

Esterase activity was performed with p-Nitrophenyl acetate (pNPA), p-Nitrophenyl butyrate (pNPB) as mentioned by Ploier B et al. (2013) [9]. Similarly for p-Nitrophenyl palmitate (pNPP) substrate, assay was conducted as per Shamsher S K et al. (2005) [10]. Controls were set with only pYES2/CT vector alone over expressed and purified under the same conditions. All the assays were performed in triplicates and mean values were recorded. Michaelis-Menten kinetics was analyzed using Graph Pad Prism version 5.

### TAG Lipase assay

TAG lipase activity was assayed using Lipase Activity Assay Kit (K722-100; Biovision, Mountain View, CA) following the manufacturer's protocol. In brief, lipase hydrolyzes the triglyceride substrate to form OxiRed probe linked glycerol, which is measured at 570nm. TAG lipase activity assay of Ymr210wp enzyme was performed with 5μl of 1.4642±0.13 mg/ml i.e., 7.32 μg of purified enzyme [11, 12].

### Separation of lipid classes

Different lipid classes were separated on LC-Silica Sep Pak cartridges (3ml, 500mg, Supelco) according to Lynch and Steponkus [13]. Appropriate amount of total lipid extracts was dissolved in 1 ml of chloroform and transferred to the Sep-Pak cartridge. The cartridge was sequentially eluted with 10 ml of chloroform for neutral lipids. After draining the first solvent, 15 ml of acetone: methanol (9:1, v/v) was added to elute the glycolipids and ceramides. Finally, 15 ml of methanol was added into the cartridge to elute phospholipids. All three fractions were dried under nitrogen purge and used immediately. Extra fractions were reconstituted with small volume of chloroform (for neutral lipid) and chloroform: methanol (2:1, v/v) (for other fractions) under nitrogen and stored at -20°C until further analysis.

### Separation of neutral lipid subclasses

Different neutral lipid subclasses were further separated on LCSilica Sep Pak cartridges (3ml, 500mg, Supelco). Appropriate amount of neutral lipid fraction from last step was re-dissolved in 1 ml of hexane. This was transferred to the Sep-Pak cartridge and sequentially added another 3 ml of hexane to elute hydrocarbons. After draining the first solvent hexane, 6 ml of hexane: diethyl ether (99:1, v/v) was added to elute steryl esters. Sequentially added another 5 ml of hexane: diethyl ether (95:5, v/v) to elute triglycerides and 5 ml of hexane: diethyl ether (92:8, v/v) to elute free fatty acid. All fractions except the hydrocarbons were evaporated under nitrogen purge and used immediately or stored by reconstituting with small volume of chloroform under nitrogen at -20°C until further FAMEs analysis.

### Preparation of FAMEs

Fatty acid methyl esters (FAMEs) were prepared by 2% H2SO4 methanol method. 2% H2SO4 in methanol was prepared by mixing 2 ml of H2SO4 with 100 ml methanol [14]. FAMEs were extracted by the addition of 2 x 2 ml aliquots of hexane and vortexing. The two layers were allowed to separate and the upper hexane layer was collected, and subjected to gas chromatography analysis for identification and quantification of fatty acids.

### Gas chromatographic analysis of FAMEs

Analysis of FAMEs was performed on Agilent 6890N gas chromatography instrument coupled with an Agilent MS-5975 inert XL mass selective detector (Agilent Technologies) in the Electron Impact (EI) mode. Separation of fatty acids was achieved by injecting 2 μL of the FAMEs on to (88% - Cyanopropyl) arylpolysiloxane column, HP88 (Agilent J & W Scientific, 30 x 0.25 mm x 0.25 μm). Split less injection was performed with a constant carrier gas (helium) at a flow rate of 1 ml/min. Inlet temperature and transfer line temperatures were set at 200°C and 180°C respectively. Temperature programming was as follows: initial isotherm of 80°C held for 1 min, raised to 90°C at the 1°C / min, 90-250°C at a rate of 6.1°C / min with a hold of 15 min at the final temperature. The MS ion source temperature was 230°C and the Quadruple temperature was 150°C. Peak identification of fatty acids in the analyzed samples was carried out by comparison of chromatogram with mass spectral library (NIST) and against the retention times and mass spectra of Supelco 37 component FAME mix (Sigma-Aldrich, St Louis, MO, USA).

### Statistical analysis

Data was analyzed through paired t-test. Level of significance was evaluated from the p-value of 0.05.

## Results

During systematic analysis of putative yeast lipase gene deletion strains, ymr210wΔ showed accumulation of lipid droplets and was characterized by its elevated levels of cellular TAG. Further, lipid profiling was done to understand the changes in the different lipid classes in the ymr210wΔ strain in comparison to WT.

### Ymr210wp shows TAG lipase activity in-vitro

To determine the hydrolytic activity, recombinant protein expressed in S. cerevisiae was purified using Ni-NTA agarose column. Ymr210wp showed TAG lipase activity of 3.26±0.31 nmole/min/mg of protein against controls.

### In vitro esterase assay confirms hydrolytic activity of Ymr210wp

**Figure 1:**
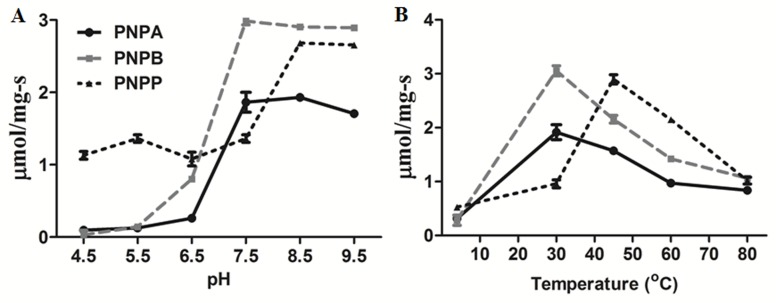
pH and Temperature optimum: (A) Represnts pH optimum and (B) Temperature optimum for Ester hydrolase activity of ymr210wp with pNPA, pNPB and pNPP substrates. The ymr210wp has optimum activity with pH 7.5 and temperature 30°C for the substrates pNPA and pNPB. Whereas, with pNPP ymr210wp showed maximum activity at pH 8.5 and temperature 45°C.

Hydrolytic activity was also assayed at different pH 4.5, 5.5, 6.5, 7.5 and 8.5 with pNPA, pNPB as well as pNPP substrates and found to be optimum at pH 8.5 for pNPP and pH 7.5 for both pNPA and pNPB substrates respectively (Figure 1A). The esterase activity was also monitored at different temperatures including 30, 45, 60 and 80°C and pNPA and pNPB were found to be optimum at 30°C whereas, pNPP substrate reaction was optimum at 45°C (Figure 1B). Ymr210wp cleaved pNPA with a Km of 11.51±2.95 mM and a Vmax of 0.26±0.03μmol/min/mg (Table 1), pNPB with a Km of 7.28±1.61mM and a Vmax of 0.18±0.01 μmol/min/mg and pNPP with a Km of 13.19±1.03mM and a Vmax of 0.33±0.14 μmol/min/mg. Enzyme tested showed reproducible hydrolytic activities with these substrates against control samples. Based on these results, Ymr210wp confirms both TAG lipase and ester hydrolase activities.

Table 1 Showing the parameters of Michaelis Menten kinetics (Km, Vmax and kcat/Km) of Ypr147cp esterhydolase with pNPA, pNPB and pNPP as substrates. The kinetics of YMR210wp depicts that pNPB has minimum Km value compare to rest of the substrate (7.28±1.61mM). Whereas, Vmax is higher for pNPP (0.33±0.05) when compare to pNPA and pNPB.

### Impact of YMR210w deletion on lipid profile

**Figure 2:**
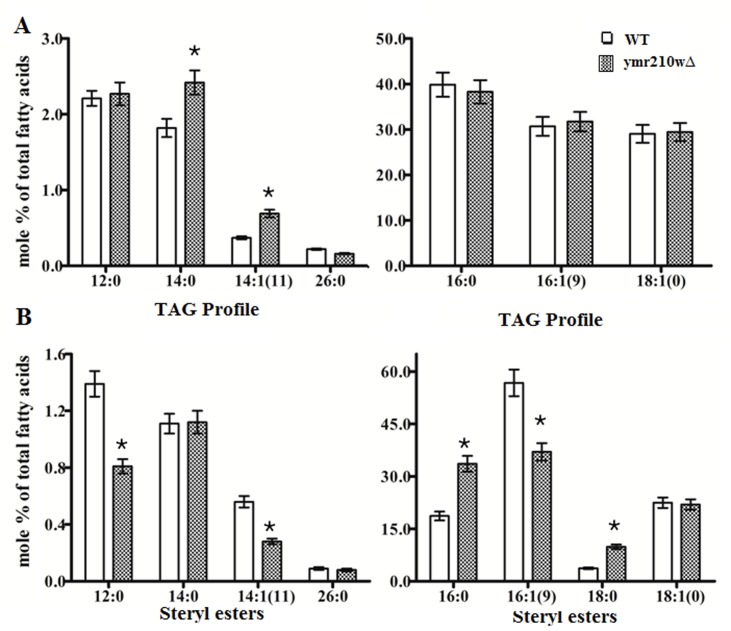
TAG and SE lipid profiles showing variations: GC-MS analysis of FAMEs was performed on Agilent 6890N gas chromatography instrument. Lipid profiles of WT and ymr210wΔ are depicted. (A) The TAG profile shows a significant increase of 14:0, 14:1(11) lipid classes in ymr210wΔ strain compared to WT. (B) The steryl esters 16:0 and 18:0 of ymr210wΔ increased compared to WT. Whereas, 12:0, 14:1(11) and 16:1(9) lipid classes were decreased ('*' represents significant difference).

Lipid profiling of ymr210wΔ clearly distinguished the accumulation of certain lipid classes. The total TAG content showed an increase of 25% (p=0.043) of 14:0 tetradecanoic acid (myristic acid) and a rise of 47% (p=0.012) of 14:1in ymr210wΔ strain when compared to WT cells by GC-MS analysis (Figure 2A). There was no significant change in the other TAGs detected (Figure 2A). Steryl esters showed significant changes with 16:0 hexadecanoic acid (palmitic acid) augmentation by 80% (p=0.014) and 18:0 octadecanoic acid (stearic acid) by 165% with p value 0.006 (Figure 2B), while compensating this 16:1(Δ9) palmitoleic acid reduced by 35% with p=0.025. Dodecanoic acid 12:0 (lauric acid), 14:1 decreased by 42 (p=0.017) and 49% (p=0.011) respectively (Figure 2B) while other lipids were not varied significantly.

**Figure 3:**
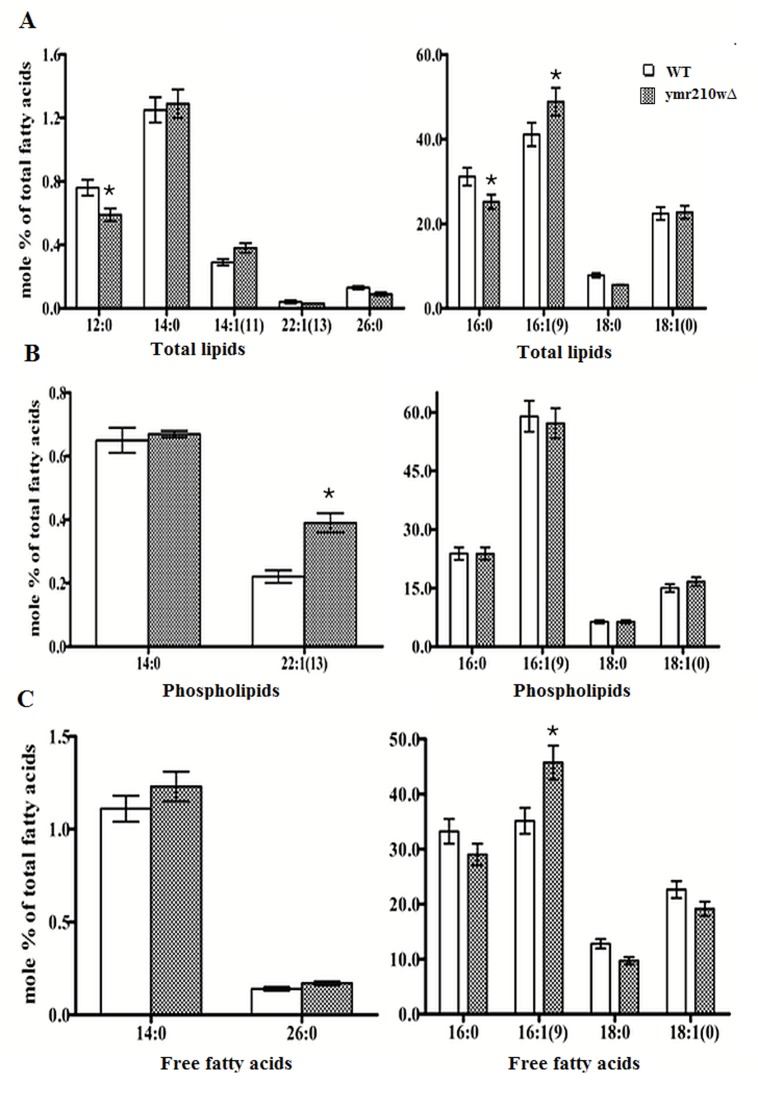
Lipid profiles estimated with GC-MS: Lipid profiles of WT and ymr210wΔ are depicted. (A) Total lipids 12:0 class significantly decreased and 16:1(9) significantly increased compare to WT. (B) Phospholipid profile of ymr210wΔ strain shows 22:1(13) increased significantly compare to WT and (C) Depicts free fatty acids of 16:1(9) significantly increased. Significance represented as '*'.

Total lipid content with 16:1(Δ9) palmitoleic acid showed only 16% increase, while there was 24% decrease (p=0.080) in 16:0 hexadecanoic acid (palmitic acid) and 29% decrease (p=0.066) in 12:0 dodecanoic acid (lauric acid) content. There was no significant change in the other total lipids detected (Figure 3A).

Phospholipids showed significantly 43% add on of 22:1 (Δ13) erucic acid with p value of 0.015, while rest of the phospholipids was not showing any significant difference (Figure 3B). Free fatty acid content did not show any significant changes except for 16:1(Δ9) palmitoleic acid, which was enhanced (p=0.060) by 23% (Figure 3C).

## Discussion

Lipases play an important role in maintaining lipid homeostasis in cells [15, 16, 17, 18]. YMR210w was previously reported as a member of three-gene family of Saccharomyces cerevisiae involved in medium chain fatty acid ethyl ester synthesis. The Ymr210wp protein sequence shows the lipase catalytic domain and it belongs to α/β hydrolase family [19]. To study the functionality of YMR210w, it was over expressed and His-tag purified recombinant protein was used for in vitro enzyme assays. There was no lipase activity reported with Eht1 and Eeb1 and both enzymes showed esterase activity with only short chain pNP substrates [6] while the present study highlights the esterase activity of Ymr210wp with both long and short chain pNP substrates. This is in agreement with the results of the cellular TAG analysis in the WT, ymr210wΔ and Δ+ strains (data not shown). Deletion of YMR210w led to an increase in the TAG content while the TAG levels in ymr210wΔ were rescued by recombinant YMR210w expression in ymr210wΔ strain justifying the role of Ymr210wp in TAG turnover elsewhere by Kandasamy Selvaraju et al. [7].

YMR210w was reported as an ortholog of Drosophila melanogaster's CG3488, which on chromosomal deletion resulted in excess lipid phenotype and was rescued by over expression [20]. This result is in line with the present data of the increased accumulation of lipid droplets, the storehouse of TAG and SE in ymr210wΔ and can be attributed to TAG lipase and ester hydrolase activities along with MAG lipase reported [7].

Ymr210wp also has the conserved HXXXD motif at C-terminal and was identified as a consensus sequence of BAHD super family of plants which participate in the biosynthesis of secondary metabolites and utilize relatively hydrophilic acyl- CoA-activated donors to catalyze acetyl-, malonyl-, benzoyl-, and hydroxycinnamoyl- transfer reactions [21]. YMR210w was previously reported to be involved in the synthesis of ethyl octanoate and ethyl deconoate and could be attributed to the acyl-transferase motif HXXXD [6].

Increased levels of myristic acid (14:0) in ymr210wΔ strain indicates the preference of myristic acid as substrate and the same type of activity was also reported with Tgl4p, the yeast ortholog of the mouse adipose triglyceride lipase (ATGL) with high specificity for TAG and preference for myristic and palmitic acid as substrates. The ymr210wΔ strain phospholipids analysis showed significant increase in the C22:1 (Δ13) erucic acid which is comparable to the activity of Tgl5p with very long chain fatty acids (VLCFA) [2, 4, 5]. YMR210w was also found to have significant impact on aroma production in a study on volatile aroma compounds and the respective candidate gene expression levels involved in aroma profile modifications. Over expression of YMR210w was positively correlated with production of ethyl acetate, ethyl caprylate and isoamyl acetate linking metabolic networks by transcriptome analysis in a comparative study of different wine yeast strains [22].

## Conclusion

In addition to MAG lipase activity, Ymr210wp also possess ester hydrolase and low but persistent TAG lipase activity. This dual functionality has led to the accumulation of steryl esters and phospholipids in ymr210wΔ leading to increased lipid droplets when compared to WT.

## Conflict of interest

The authors declare that there is no conflict of interest.

## Role of Funding

This work was fully supported by Council of Scientific & Industrial Research (CSIR), (09/1068(0001)/EMR-I/2011), Ministry of Science and Technology, Govt. of. INDIA. University Grants Commission (UGC), (F.30-1/2013 (SA-II)/RA-2012-14-GEANP- 1237), Govt. of. INDIA.

## Figures and Tables

**Table 1:** Kinetic parameters of YMR210wp

Substrate	Km (mM)	Vmax (s-1)	kcat/Km (mM-1s-1)
pNPA	11.51±1.09	0.26±0.03	117
pNPB	7.28±1.61	0.18±0.03	128
pNPP	13.19±1.03	0.33±0.05	130

## Abbreviations

TAG: triacylglycerol
LD: lipid droplet
w/v: weight/volume
v/v: volume/volume
Px: peroxisome(s)
SE: steryl ester(s)
TEM: transmission electron microscope
pNP: p-nitrophenyl
pNPA: p-nitrophenyl acetate
pNPB: p-nitrophenyl butyrate
pNPP: p-nitrophenyl palmitate
FAMEs: Fatty acid methyl esters
